# Transformation of glass fiber reinforced epoxy from waste printed circuit boards to adsorbents for effective CO_2_ storage in abandoned mines

**DOI:** 10.1039/d6ra00139d

**Published:** 2026-01-21

**Authors:** Jacob Rubel, Pavla Eliášová, Martin Kubů, Yukun Ji, Veerle Vandeginste

**Affiliations:** a KU Leuven, Campus Brugge, Department of Materials Engineering Bruges 8200 Belgium veerle.vandeginste@kuleuven.be; b Charles University, Faculty of Science, Department of Physical and Macromolecular Chemistry Hlavova 8 Praha 2 12840 Czech Republic pavla.eliasova@natur.cuni.cz; c China University of Mining and Technology, State Key Laboratory for Geomechanics and Deep Underground Engineering Xuzhou 221116 China jykcumt@163.com; d Yunlong Lake Laboratory of Deep Underground Science and Engineering Xuzhou 221116 China

## Abstract

Waste printed circuit boards (PCBs) pose environmental challenges due to their toxic and varied composition. Current recycling methods prioritize metal recovery, leaving non-metallic fractions (NMFs) underutilized. These NMFs can be converted into nitrogen-doped porous carbons with good CO_2_ adsorption capacity. However, waste PCBs are dominated by the FR-4 type in which fibers comprise a large mass fraction and intrinsically limit carbon yield and accessible porosity. We report a targeted valorization route for FR-4 NMFs that couples one-step KOH/urea activation with post-treatments aimed at the glass-fiber/polymer composite: (i) aqueous ultrasonication to increase the surface area by enhancing surface roughness and fragmenting particles to smaller sizes, and (ii) amine functionalization with polyethylenimine (PEI) to improve CO_2_ affinity. Ultrasonication significantly increased BET surface area (from 444 to 928 m^2^ g^−1^) and CO_2_ uptake (from 1.34 to 1.90 mmol g^−1^), primarily through particle size reduction. PEI functionalization increased isosteric heat of adsorption (indicative of stronger CO_2_ affinity) but concurrently caused pore blockage, lowering net adsorption capacity. Quantitative analysis across all samples shows that CO_2_ uptake correlates strongly with microporosity and total BET area, weakly with external surface area. We discuss mechanistic roles of cavitation-driven fragmentation and glass-fiber/carbon interplay. Our findings suggest ultrasonication is a promising method for enhancing PCB-derived porous carbons in carbon capture applications, while PEI functionalization needs further optimization to balance affinity and accessibility.

## Abstract 2

Ultrasonication enhances surface area and CO_2_ uptake of FR-4 PCB-derived porous carbons, while PEI functionalization increases CO_2_ affinity with porosity trade-offs.

## Graphical abstract

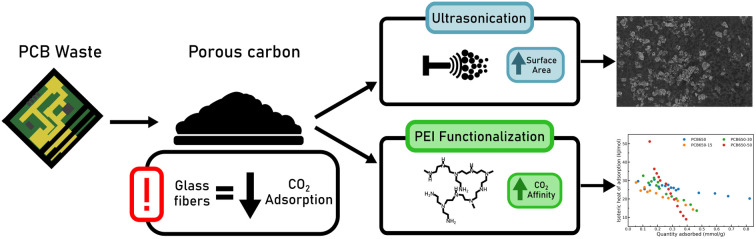

## 1. Introduction

Printed circuit boards (PCBs) are the basis of electronic devices across the world. The past decades have seen a strong increase in the production of electronic devices, and more devices incorporate electronics into their framework. However, at their end of life, PCBs become a hazardous and difficult to dispose of waste material. Most of the metallic fraction of PCBs can be physically and/or chemically removed and directly recycled as a high-value product.^1^ In contrast, the bulk of PCBs mostly consists of non-metallic materials which are not easily recycled. FR-4 PCBs, the most common type of rigid PCBs, which are used in a variety of consumer electronics, are composed of a fiberglass weave impregnated with a fire resistant epoxy resin, which cannot be recycled back into its original components. Instead, these non-metallic fractions (NMFs) of PCB waste must be disposed of in a landfill or *via* incineration, resulting in hazardous by-products such as dioxins, furans, polybrominated organic pollutants and polycyclic aromatic hydrocarbons.^2–4^ In many PCBs, NMFs account for 70–90% of PCB waste by mass.^5,6^ Hence, it is favorable to develop new recycling methods for these materials to better protect the environment and limit pollution.

There are three primary paths for dealing with the NMFs: incineration, composite manufacturing, and pyrolysis. Incineration should be avoided due to the toxic byproducts it produces, but composite manufacturing and pyrolysis allow for PCB waste to be converted into useful products. Valuable composites containing polymers such as polyamide^7^ and polyvinyl chloride^8^ as well as concrete^9^ can be made using the NMFs. This is because most NMFs (specifically the most common FR-4 type) contain glass fibers, a typical reinforcing material, and flame retardant resins. Therefore, as filler materials PCB NMFs can impart greater mechanical strength and heat resistance to their composite materials, enhancing overall performance.^7^ However, pyrolysis processes have shown the potential to have a more positive environmental impact by producing products which suppress the generation of more pollutants compared to their conventional production paths *versus* composite manufacturing.^10^ Thus, new pyrolysis and pyrolysis post-treatment processes would be beneficial for improving the environmental friendliness of the PCB waste treatment process.

The pyrolysis of NMFs waste involves careful heating in the absence of oxygen, often along with a chemical agent, to produce gasses, oils, and porous carbons.^11^ Amongst these products, porous carbons have the greatest versatility and can be used as gas adsorbents,^12^ water treatment filters,^13^ and electrode materials.^14^ A promising method for producing porous carbon from PCB waste is through the use of KOH as a chemical activation agent. Studies by Ke *et al.* and Kan *et al.* have shown that it is possible to achieve specific surface areas of 3112 m^2^ g^−1^ and 2792 m^2^ g^−1^, respectively.^12,15^ However, these studies used FR-3 type PCBs which contain more resin and fewer glass fibers than the more prolific FR-4 type. Moreover, the porous carbon network can be modified through processes such as nitrogen doping. Cao *et al.* demonstrated this through the use of ionic liquid modification to introduce N and S to the porous network to produce porous carbons for their application as an oxygen reduction catalyst.^16^ In our previous study, we also showed how urea can be used as a nitrogen source to dope porous carbons produced from NMFs using KOH as a chemical activation agent.^17^

A particularly valuable application of porous carbon derived from PCB waste lies in its ability to act as a carbon sequestration material. By doping the produced carbon material with nitrogen, a CO_2_ selective adsorbent can be created.^18^ This would allow for the adsorption of CO_2_ into the porous carbons which could then be sequestered underground in locations such as abandoned mines. Such processes are especially needed as the potential storage capacity of abandoned mines is very high with around 7.2 billion cubic meters in China alone.^19^ High CO_2_-capacity materials such as nitrogen doped porous carbons are imperative in maximizing the use of this space. Thus, making porous carbons from non-metallic PCB waste serves a dual purpose in disposing of an inconvenient waste material while simultaneously removing CO_2_ from the atmosphere combating anthropogenic climate change. However, there is still limited research into making this process a reality. To the best of our knowledge, our own previous work is the only study focused on turning the most ubiquitous form of PCB (the FR-4 type) into porous carbon for this purpose.^17^

In contrast to resin-rich FR-3 precursors commonly used in earlier PCB-to-carbon studies, FR-4 NMFs require tailored processing to overcome low CO_2_ affinity of the inorganic glass component while protecting the carbon pore network. Building on our previous work on KOH/urea activation of FR-4 NMFs,^17^ we focus explicitly on strategies that act on the composite rather than the carbon phase alone. Here, we introduce a dual post-treatment designed for FR-4-derived materials: (i) ultrasonication of carbonized PCB samples in water, leveraging microbubble cavitation to increase the surface roughness of silicate glass and fragment both glass fibers and carbon;^20^ and (ii) polyethylenimine (PEI) functionalization, exploiting amine groups to increase CO_2_ affinity on glass-fiber surfaces and at carbon sites as demonstrated in mesoporous silica and carbon.^21,22^ Our central hypothesis is that combining physical fragmentation (to increase surface area) with amine chemistry (to enhance affinity) can improve capture performance in FR-4-derived CO_2_ adsorbents where the glass fraction otherwise dilutes capacity. We show that ultrasonication is effective for this feedstock, and that PEI requires careful loading control to avoid pore blocking.

## 2. Materials and methods

### 2.1. Materials

The FR-4 type waste PCBs used in this study were obtained locally from teaching electronics projects at KU Leuven-Brugge. The boards were separated from the electronics to obtain mostly non-metallic PCB waste. The bulk of their mass consisted of a glass fiber and flame retardant epoxy composite with small amounts of copper coated on the surface. Urea (99.5%, for analysis) was purchased from Thermo-Scientific. KOH (analytical reagent grade) and HCl (37% concentrated, analytical reagent grade) were both purchased from Fisher Scientific. Polyethylenimine (branched, average Mw ∼800 g per mol by LS) was purchased from Fluorochem.

### 2.2. Preparation of functionalized porous carbons

Porous carbon was produced from PCB waste following the same procedure as our previous work.^17^ Briefly, PCBs were ground and sieved to <200 µm and heated in a one-step procedure with KOH and urea to produce nitrogen doped porous carbons. This was done with a 2 : 1 : 1 KOH : urea : PCB ratio mixture which was heated in a horizontal quartz tubular furnace Nabertherm model RSH 50/500/13 with controller P580 (Nabertherm, Germany) to 450 °C at a rate of 5 °C min^−1^ where it was held for 1 hour. Immediately after this, the sample was heated at 5 °C min^−1^ to an activation temperature of 650 °C. The product was allowed to cool to room temperature before being extracted from the furnace and treated with HCl to neutralize any excess KOH. This product was called PCB650 and was the base material for all subsequent samples.

For the non-ultrasonicated samples, the PCB650 was washed twice with DI water and once with ethanol before being dried in an oven at 40 °C overnight. For ultrasonicated samples, the PCB650 was washed twice with DI water then dispersed in fresh DI water and ultrasonicated using a QSONICA Q700 ultrasonic probe at approximately 80–90 W and 22 Hz for 1 hour. After this, these samples were also washed once with ethanol before drying overnight at 40 °C to produce samples designated with US-PCB650.

For PEI functionalization, the PCB650 and US-PCB650 samples were treated following the same procedure based on PEI functionalization methods for mesoporous silica.^21,22^ First, the selected amount of PEI was dissolved in 0.791 g of methanol and stirred for ∼15 min. After this 0.2 g of PCB650 or US-PCB650 was added to the solution to form a slurry which was stirred for 30 min. After 30 min, stirring was stopped and the slurry was dried under vacuum at 70 °C overnight. The dried functionalized PCB samples were then analyzed. The amount of PEI was varied based on the mass ratio to the amount of (US-)PCB650 with ratios of 15%, 30% and 50% being tested corresponding to the sample names: (US-)PCB650-15, (US-)PCB650-30, (US-)PCB650-50 respectively.

### 2.3. Characterization methods

The functionalized (US-)PCB650-X samples and PEI were characterized using attenuated total reflectance–Fourier transform infrared spectroscopy (ATR-FTIR) Bruker model ALPHA II compact FTIR. X-ray diffraction (XRD) analysis was done using a Bruker D2 Phaser with Cu tube source (*λ* = 0.154 nm) over a range of 0–80° 2*θ*. Elemental analysis was performed by inductively coupled plasma mass spectrometry (ICP-MS; Agilent 7900 ICP-MS). Scanning electron microscopy (SEM) images were obtained using a Thermo Fisher Scientific Scios 2 DualBeam FIB-SEM equipped with a Schottky-type field emission gun. Sample morphology was analyzed with the Everhart–Thornley secondary electrons detector, retractable backscattered electrons detector, in-lens backscattered electrons Trinity Detector T1, and in-lens secondary electrons Trinity Detector T2. Elemental composition analysis was conducted using energy-dispersive X-ray spectroscopy (EDX) using the Thermo Fisher Scientific UltraDry X-ray detector integrated into the SEM system. Data collection was performed at an accelerating voltage of 15.0 kV, and a working distance of 5.6846 mm. All samples were mounted on conductive carbon tape attached to the SEM aluminum stub holder.

Argon adsorption/desorption isotherms were measured on a Micromeritics 3Flex volumetric Surface Area Analyzer at −186 °C to determine surface area, pore volume and pore size distribution. Before measurements, all samples were degassed under the turbomolecular pump vacuum using a Micromeritics Smart VacPrep instrument; starting at an ambient temperature up to 110 °C with a heating rate 1 °C min^−1^ until the residual pressure of 13.3 Pa was achieved. After heating at 110 °C for 1 h, the temperature was increased to 250 °C (1 °C min^−1^) and maintained for 8 h. The specific surface area (*S*_BET_) was calculated by the BET method^23,24^ using adsorption data in the range of a relative pressure *p*/*p*_0_ = 0.05–0.20. The *t*-plot method^23,24^ was used to evaluate external surface area (*S*_ext_). The adsorbed amount at relative pressure *p*/*p*_0_ = 0.95 reflects the total adsorption capacity (*V*_tot_). The micropore volume (*V*_mic_) and pore size distribution (PSD) were calculated from argon isotherms by the NLDFT model for Ar@87 on carbon slit pores. The CO_2_ adsorption analysis was also done using a Micromeritics 3Flex Adsorption Analyzer and the CO_2_ isotherms were determined at four different temperatures ranging from 0 to 60 °C. Before each adsorption experiment, the samples were outgassed (250 °C for 8 h) under a turbomolecular pump vacuum using a Micromeritics Smart Vac Prep instrument until the residual pressure of 0.1 torr was achieved. A custom made thermostat was used to maintain a temperature of the sample, with an accuracy of ±0.1 °C, at 0, 20, 40, and 60 °C.

## 3. Results and discussion

### 3.1. Structure and chemical composition

**Fig. 1:**
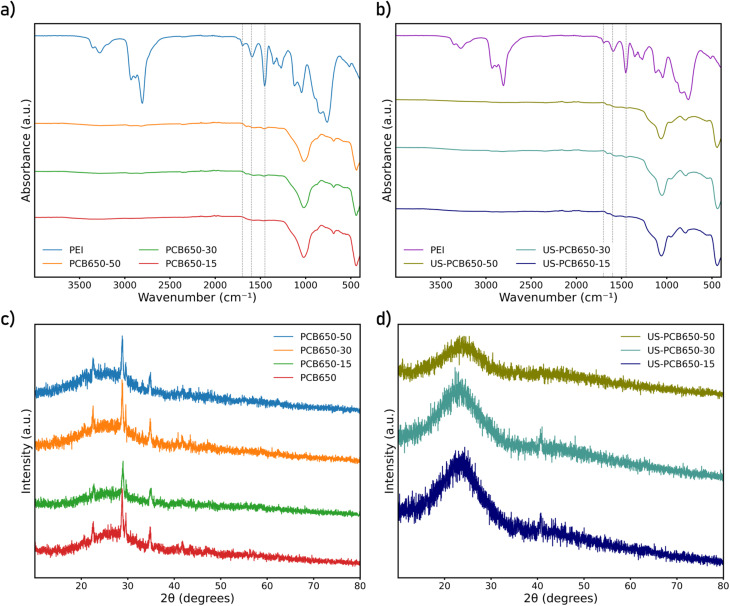
FTIR spectra (a and b) and XRD patterns (c and d) of modified porous carbon made from PCB waste.

FTIR spectra for all samples are presented in (Fig. 1a and b) along that of PEI. In the carbon samples, the spectra are similar to those from our previous study with overlapping peaks at 1040 cm^−1^ and 1000 cm^−1^ corresponding to C–N stretching in the N-doped carbon and –Si–O–Si– bonds from the glass fibers, respectively.^17,25^ The PEI spectrum matches with those found in the literature and shows peaks corresponding to C–N stretching between 1020-1250 cm^−1^; N–H stretching for aliphatic primary and secondary amines between 3300 cm^−1^ and 3400 cm^−1^; N–H bending around 1600 cm^−1^; C–H alkane stretching from 2800–3000 cm^−1^; C–H alkane bending at 1450 cm^−1^; and N–H deformation and wagging from around 700–800 cm^−1^.^26,27^ In the carbon samples, most of the PEI peaks are overshadowed by the more prominent nitrogen-doped carbon and glass fiber peaks. However, the PEI peaks at around 1450 cm^−1^, 1600 cm^−1^, and 1700 cm^−1^ do appear in the treated PCB650 samples and are absent in untreated PCB650 sample, indicating the presence of PEI within the functionalized samples. The same phenomenon is seen in the US-PCB650-X samples with the same small but notable peaks in the 1400–1700 cm^−1^ range.

XRD patterns for each of the samples can be found in (Fig. 1c and d). All samples show a similar broad peak around 23° which is attributed to the (002) plane of carbon.^28^ The US-PCB650 samples all show no other noticeable peaks over the range examined which indicates there are no other significant ordered phases in the sample. However, some additional peaks can be identified in the non-ultrasonicated PCB650-X samples at approximately 22°, 29°, and 35° which do not appear in the US-PCB650-X samples. These peaks most closely correspond to those of urea, however, due to the small size of the remaining urea crystals, the peaks are significantly weaker and broader.^29^ This would indicate that for these samples some excess urea was not completely removed from this batch of PCB650 and the additional ultrasonication step better facilitated its dissolution and removal into the DI water suspension solution.

In order to analyze the elemental composition of the porous carbons, the samples were examined through inductively coupled plasma (ICP) analysis. The full results of the analysis are given in Table 1. A notable amount of copper is present in all samples which was expected based on the source material used to make the porous carbons. PCB650-15 shows an abnormally high amount of copper compared to the other samples, however, due to the various sizes of the copper particles, it is likely that the number of copper particles may vary a bit between the different powder samples, leading to slight differences in measured copper content. A similar phenomenon was seen in our previous work in the XRD patterns of the samples with some samples showing peaks for copper and others not.^17^ Other metals such as Ni, Pd, and Sn appear in trace amounts (<0.1 mg g^−1^), all most likely leftover from the metal separation process of the PCB waste. They were either embedded in the board itself as part of the soldering process or were impurities in the copper also found in the boards. Neither ultrasonication nor PEI treatment appear to have a noticeable effect on the presence of these trace metals.

**Table 1:** Chemical composition of modified porous carbon made from PCB waste analyzed based on ICP-MS results

Sample	Al [mg g^−1^]	Si [mg g^−1^]	Ni [mg g^−1^]	Cu [mg g^−1^]	Pd [mg g^−1^]	Sn [mg g^−1^]
PCB650	39.2	176	0.064	46.7	0.014	0.022
PCB650-15	35.3	182	0.027	82.7	0.008	0.063
PCB650-30	34.2	142	0.028	29.7	0.061	0.013
PCB650-50	30.3	158	0.017	29.9	0.028	0.008
US-PCB650	—	204	0.052	28.8	0.049	0.010
US-PCB650-15	—	209	0.054	31.0	0.029	0.012
US-PCB650-30	—	194	0.045	29.1	0.004	0.009
US-PCB650-50	—	187	0.045	24.2	—	0.005

**Fig. 2:**
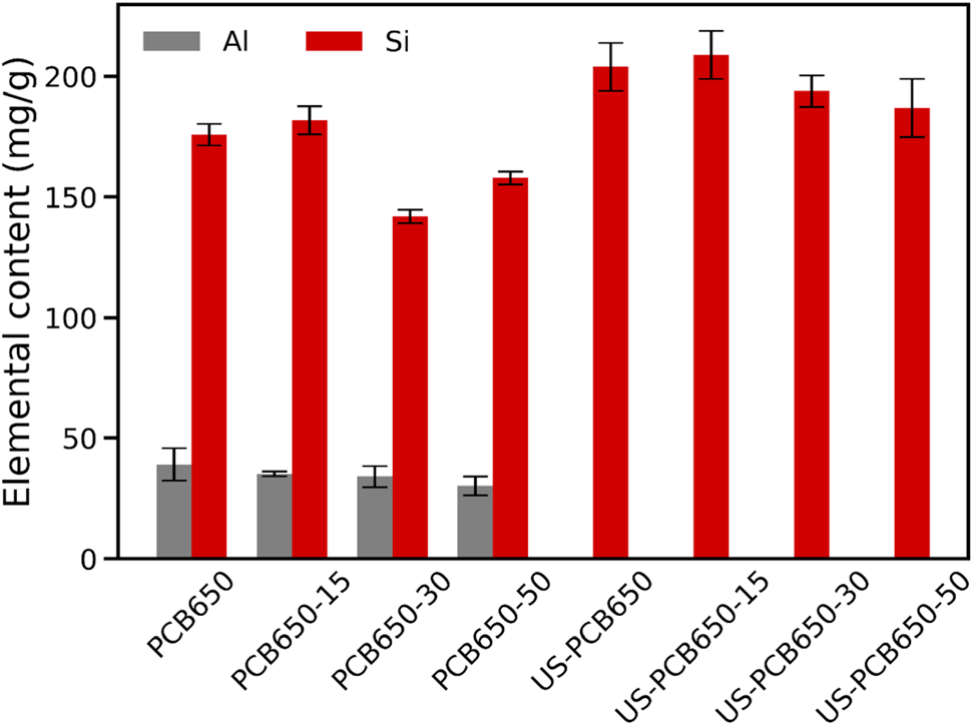
Levels of aluminum and silicon in porous carbons made from PCB waste.

Aluminum and silicon content, on the other hand, do show significant changes between the ultrasonication and non-ultrasonicated samples (Fig. 2). These two elements come from the glass fibers present in the PCB waste before treatment. Aluminum makes up a smaller portion of the glass fibers than Silicon and appears to be reduced to an undetectable level in the samples treated by the ultrasonication procedure. This would imply a significant break down of the glass fibers to near complete removal of Al from the sample. Si levels are also higher in the ultrasonicated samples. It is important to mention that carbon is not measured by ICP-MS due to its high ionization energy and strong background interferences. Therefore, any change in its mass in the sample affects the reported concentrations of other elements, which are expressed in mg per gram of the whole sample. Thus, the changes in Al and Si content indicate that some portion of the carbon is lost through the ultrasonication process.

### 3.2. Surface morphology

**Fig. 3:**
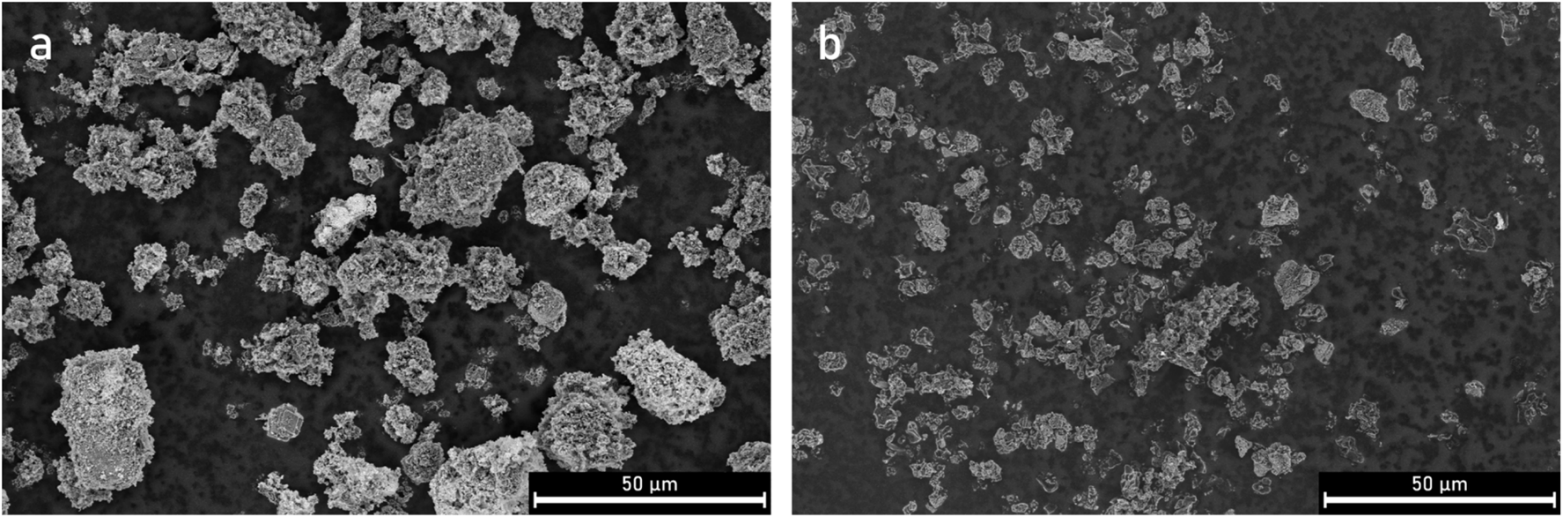
SEM micrographs of porous carbon samples PCB650 (a) and US-PCB650 (b).

**Fig. 4:**
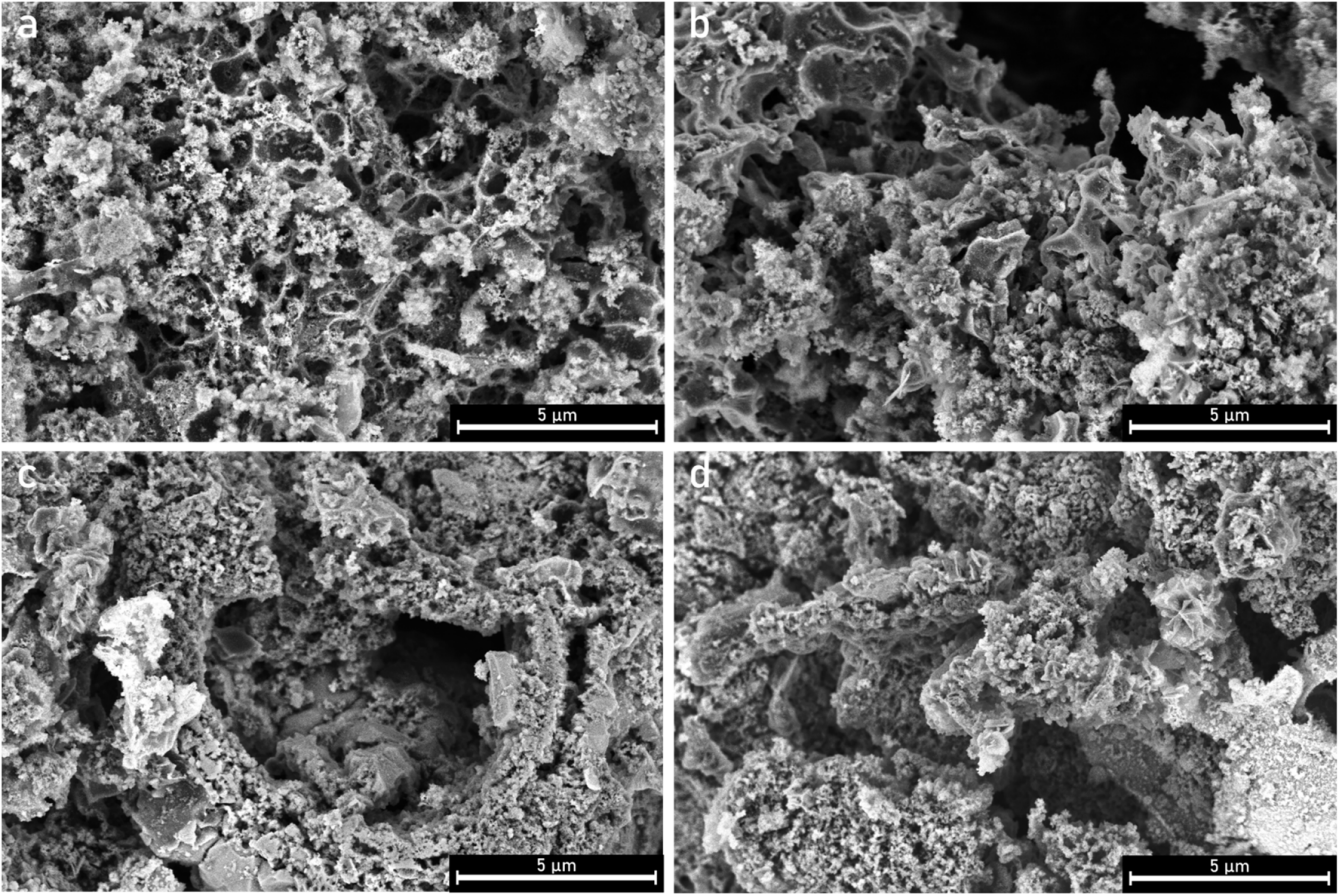
SEM micrographs of porous carbon samples (a) PCB650, (b) PCB650-15, (c), PCB650-30, and (d) PCB650-50.

The surface morphologies of the samples were analyzed using SEM. There is a clear effect of the ultrasonication on the particle size when comparing parent PCB650 and sonicated US-PCB650 (without any PEI modification), SEM images are shown in Fig. 3. The non-ultrasonicated samples (Fig. 4) all display similar surface features which match those of other porous carbons produced from PCB waste.^17,25^ All samples show evidence of the spherical macropores which are typical of porous carbons.^16,30,31^ However, the structure of these macropores is highly disrupted by the presence of the glass fibers resulting in a rough and irregular surface texture across all samples. These samples also show large amounts of small particles on their surfaces which are most likely the excess urea detected by the XRD as discussed above.

**Fig. 5:**
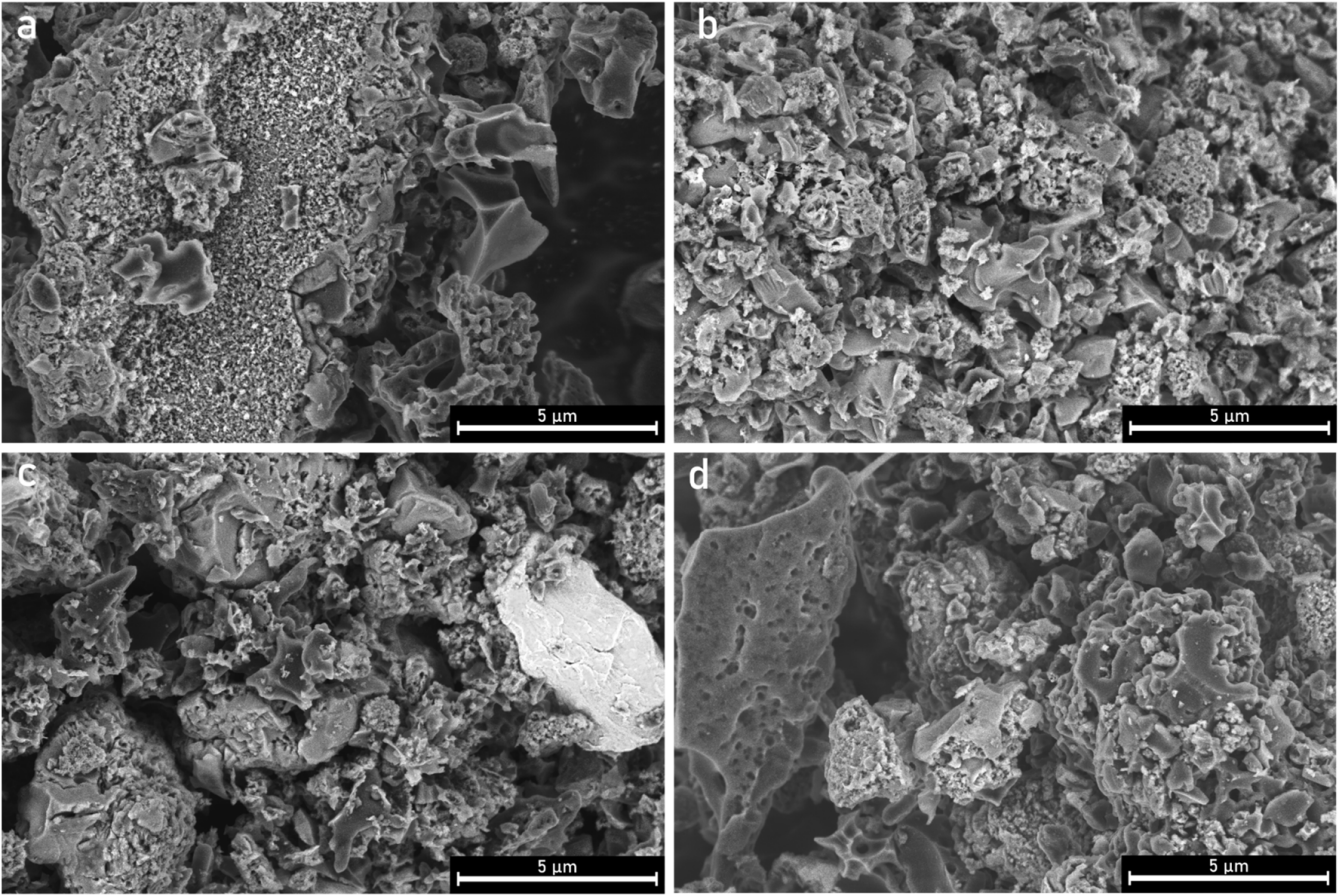
SEM micrographs of porous carbon samples (a) US-PCB650, (b) US-PCB650-15, (c) US-PCB650-30, and (d) US-PCB650-50.

The ultrasonicated samples shown in Fig. 5 have a similar surface morphology as the non-ultrasonicated samples but without the small urea particles on their surfaces. This indicates that the structure of the porous carbons was not significantly affected by ultrasonication and the porous carbon network was preserved. However, ultrasonication does seem to have a notable effect on particle size as can be seen in Fig. 3. Many particles in the PCB650 sample have a characteristic diameter of 30–40 µm, whereas all particles in US-PCB650 appear to be less than ∼15 µm. Therefore, while not directly impacting the surface morphology, ultrasonication does appear to have broken up larger carbon particles and aggregates. Notably, there are no major structural or morphological changes in the sample based on PEI loading.

**Fig. 6:**
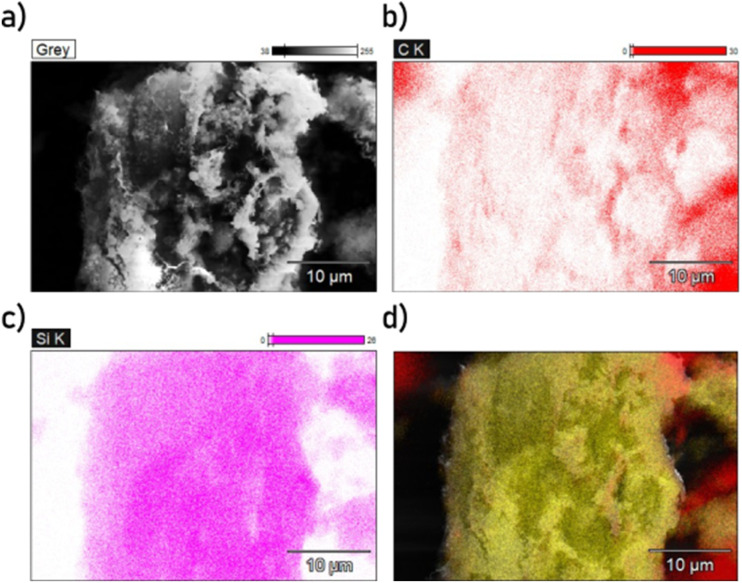
SEM-EDX mapping of PCB650 with plain SEM image (a), carbon elemental map (b), silicon elemental map (c), and Si (yellow) and C (red) overlay (d).

**Fig. 7:**
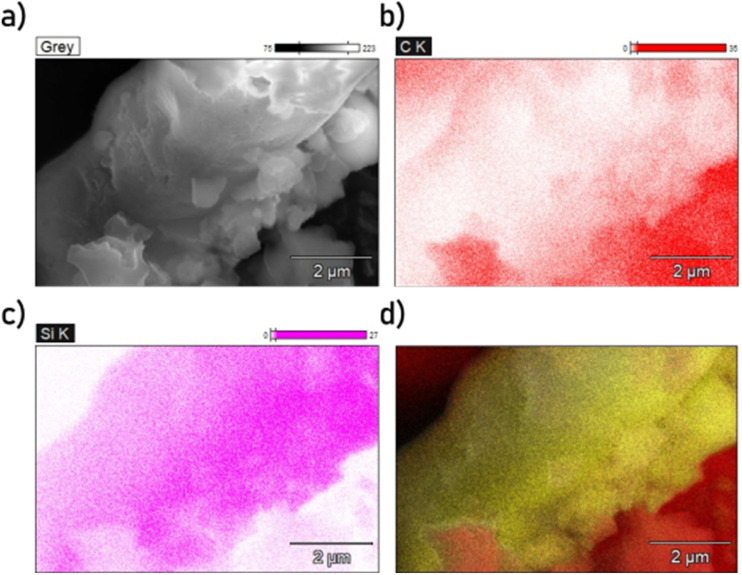
SEM-EDX mapping of US-PCB650 with plain SEM image (a), carbon elemental map (b), silicon elemental map (c), and Si (yellow) and C (red) overlay (d).

The presence and locations of the glass fibers in the samples can be determined through EDX as shown in Fig. 6 and Fig. 7. In these images, it is apparent the morphology of the glass fibers is significantly altered by the carbonation and activation process, with some large pieces containing Si bearing little resemblance to a glass fiber shape. Between the ultrasonicated (US-PCB650-X) samples and non-sonicated ones (PCB650-X), it is difficult to observe any difference in the surface texture of the glass fiber material. Therefore, without being able to measure the glass fiber surface profiles directly, it would seem that the main effect of ultrasonication was the breaking the glass fibers and carbon particles into smaller pieces as shown by the SEM images. However, this still allows significantly more of the surface of both materials to be exposed, increasing available surface area and CO_2_ capacity as will be discussed further.

### 3.3. Adsorption properties

**Fig. 8:**
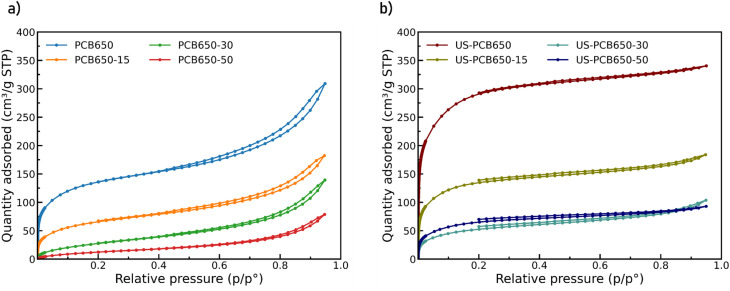
Ar adsorption isotherms for modified porous carbons produced from PCB waste using the method (a) without, and (b) with ultrasonication.

The texture properties were evaluated by argon adsorption. All samples show a Type I(b) isotherm (Fig. 8) according to the IUPAC classification with slight hysteresis (namely H4) caused by interparticle adsorption.^24,32^ The values for PCB650 are similar as in our previous study: BET surface area of 441 m^2^ g^−1^, micropore volume of 0.19 cm^3^ g^−1^), and CO_2_ capacity of 1.3 mmol g^−1^.^17^ Ultrasonication yields a consistent and substantive improvement, with US samples possessing significantly higher surface areas compared to their non-sonicated analogues (Table 2). The BET surface area increased more than twice (from 444 to 928 m^2^ g^−1^) and also the volume of micropores was notably enhanced (from 0.15 to 0.36 cm^3^ g^−1^).

**Table 2:** Ar and CO_2_ adsorption data for modified porous carbons produced from PCBs. Where BET is the Brunauer–Emmett–Teller surface area, *S*_ext_ is the external surface area calculated *via* t-plot method, *V*_mic_ is the total volume of micropores (<2 nm) calculated *via* a non-local density functional theory model, *V*_tot_ is the calculated total pore volume at *p*/*p*_0_ = 0.95, and CO_2_ uptake was measured at 750 Torr and 293K

Sample	BET (m^2^ g^−1^)	*S*_ext_ (m^2^ g^−1^)	*V*_mic_ (cm^3^ g^−1^)	*V*_tot_ (cm^3^ g^−1^)	CO_2_ Uptake (mmol g^−1^)
PCB650	444	160	0.15	0.39	1.34
PCB650-15	216	118	0.07	0.23	0.58
PCB650-30	95	95	0.02	0.18	0.54
PCB650-50	45	45	—	0.10	0.35
US-PCB650	928	78	0.36	0.43	1.90
US-PCB650-15	451	63	0.16	0.23	1.09
US-PCB650-30	173	59	0.06	0.13	0.9
US-PCB650-50	213	39	0.08	0.12	0.86

**Fig. 9:**
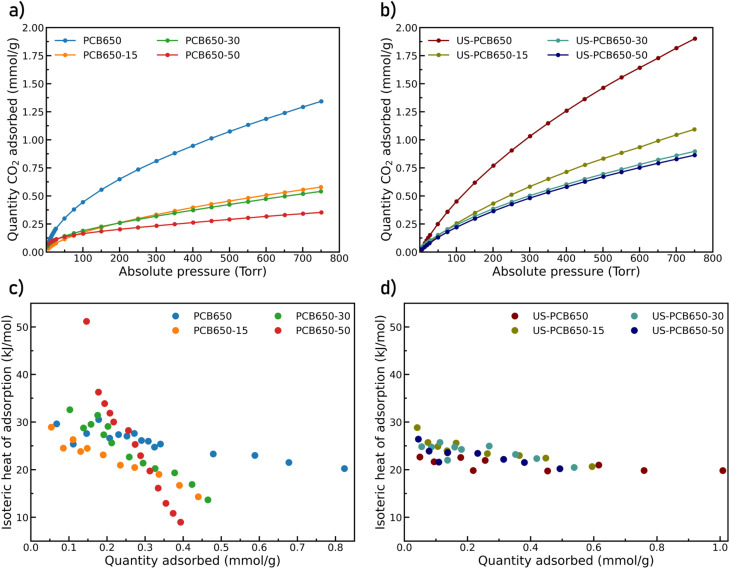
CO_2_ adsorption isotherms at 293K (a and b) and isosteric heat of CO_2_ adsorption plots (c and d) for modified activated carbons produced form PCBs.

CO_2_ adsorption isotherms at 293K for all samples are given in (Fig. 9a and b). The CO_2_ uptake capacities of the samples are comparable to those of other activated carbons which range from approximately 1 to 6 mmol g^−1^.^18,30,33,34^ US-PCB650 (without any PEI modification) showed 42% increase of CO_2_ uptake (1.90 mmol g^−1^) in comparison with the parent PCB650 (see Table 2). This is in alignment with argon adsorption data where both BET surface area and pore volumes increased after ultrasonication. These values are high within the FR-4 context, where glass fibers dilute the carbon phase; our study intentionally targets improvement for material derived from waste FR-4 type PCBs rather than from resin-rich and agricultural precursors. Most activated carbons in the literature are made from agricultural wastes which are rich in carbon and low in other impurities compared to FR-4 type PCB waste. Activated carbons produced from other industrial waste such as spent ion-exchange resin tend to show lower CO_2_ capacities of, for example, 0.8 mmol g^−1^.^35^ Overall, the ultrasonication step has a strong effect on the textural properties - the particle sizes were decreased (as evidenced by SEM images) and consequently the surface area increased and the hysteresis loops indicating the presence of interparticle voids diminished.

PEI functionalization introduces a trade-off. After the PEI loading, we observed the decrease in both BET surface area and microporosity as the added PEI caused some blocking of available pores, lowering the overall accessible surface area. This trend was found for both the non-US and US series being more significant for the non-US series. Similarly, the PEI modification led to the decrease in CO_2_ uptake in both series with the same trend–the higher PEI loading, the lower CO_2_ uptake. However, isosteric heat of adsorption increases with added PEI at low loadings (see below).

The decrease in BET surface area is not a wholly unexpected result, as some other studies on PEI functionalization with both pure silica and pure porous carbon materials show a moderate to large reduction in total surface area samples after functionalization with PEI. In two studies by Han *et al.* and Xu *et al.*, porous silica after functionalization with PEI at a ratio of 50% showed a drop in surface area from 261 to 1.4 m^2^ g^−1^ and 1480 to 4.2 m^2^ g^−1^ respectively.^21,22^ However, in both studies, the samples still showed good CO_2_ adsorption capacities of 2.6 and 1.0 mmol g^−1^ respectively. In studies on porous carbon, as the one by Liu *et al.*, a similar phenomenon is observed.^36^ Here, activated carbon fiber is functionalized with PEI at a ratio of 40% and shows a drop in BET surface area from 573 to 533 m^2^ g^−1^, notably less dramatic than the silica-based samples. Again, the PEI functionalization here led to an improvement in CO_2_ adsorption capacity from 1.46 to 2.45 mmol g^−1^ with PEI. Nevertheless, the authors of this study did report that adding higher amounts of PEI (60% ratio) did begin to reduce CO_2_ adsorption capacity again. This is significantly higher than the lowest ratio used in this study which still shows a negative impact on CO_2_ capacity. Given the greater structural susceptibility of our FR-4-derived material, optimization windows will likely lie at lower PEI fractions than those typical for mesoporous silica.

Further CO_2_ adsorption tests were performed at 273K, 313K, and 333K and used to calculate the isosteric heat of adsorption (*Q*_st_) for each sample based on the Clausius Clapeyron equation.^23^ In the figure, *Q*_st_ is plotted as positive by convention despite being negative value. Therefore, a higher *Q*_st_ value corresponds to a greater amount of heat released by the adsorption of a CO_2_ molecule onto the surface and therefore a greater affinity for CO_2_. These data are plotted in (Fig. 9c and d) *versus* quantity of CO_2_ adsorbed. The non-US samples show a slightly higher value for the isosteric heat of adsorption, especially at lower adsorbed amounts, indicating a stronger affinity for the adsorption of CO_2_ at the surface of the material. This could be due to small copper impurities (∼30 mg g^−1^ according to ICP results) which were broken up and dissolved into the water in which the ultrasonicated samples were suspended. It is also possible that a similar process happened for the nitrogen doped carbon network where bonds at high-energy nitrogen sites were more easily broken by cavitation of bubbles in water. These nitrogen rich particles then became too small to recover from the solution through centrifugation and were subsequently lost from the samples, reducing the number of CO_2_ adsorption sites. With increasing PEI loading the dependence of the *Q*_st_ becomes steeper. In the case of US samples, we do not observe such a clear trend. It is important to emphasize that there are two types of CO_2_ adsorption sites – one provided by porous carbon and the other one provided by anchored PEI polymer molecules. The contribution of each site to the overall adsorption capacity of the material cannot be easily distinguished. A study dedicated to XPS and elemental analysis could be conducted in the future to collect more detailed information on nitrogen speciation; here, we have derived the affinity enhancement through FTIR signatures and *Q*_st_ trends.

In summary, the highest CO_2_ adsorption capacity was observed for ultrasonicated PCB650 without PEI modification, 1.90 mmol g^−1^. The functionalization of the porous carbon with PEI polymer did not show the expected enhancement of CO_2_ adsorption. This is most probably due to the partial blockage of the adsorption centers of the porous carbon by PEI species. This adverse effect prevails over the inherent ability of the polymer to provide adsorption sites. Nevertheless, the ultrasonication step itself was sufficient enough to further improve CO_2_ adsorption for 42% (up to 1.9 mmol g^−1^) which significantly surpasses the previous results obtained for this type of material.^17^

### 3.4. Mechanistic interpretation: ultrasonication and glass-fiber/carbon interplay

The SEM comparison (Fig. 3) demonstrates a characteristic reduction in particle size (30–40 µm to <15 µm) after ultrasonication without discernible damage to the carbon macropore architecture (Fig. 4 and 5), indicating cavitation-induced fragmentation. The ICP trends (Fig. 2 and Table 1) further show Al depletion to below detection and an increase in Si concentration in ultrasonicated samples, consistent with preferential loss of small carbonaceous fragments during washing and with enhanced exposure of the glass component. Together, these observations support a mechanism of area exposure by fragmentation; the accessible surface is raised, and more of the composite interface becomes available for physisorption.

We connect these structural changes to texture evolution: argon physisorption (Fig. 8) transitions to larger BET and higher micropore volume (0.15 to 0.36 cm^3^ g^−1^), while the interparticle hysteresis (H4) diminishes, consistent with reduced aggregate voids. To quantify the structure–performance relation, we analyzed correlations across all samples between CO_2_ uptake (293 K, 750 Torr) and textural metrics. This analysis indicates that micropore volume and total area are the principal drivers of capacity in FR-4-derived carbons, whereas external area contributes little, aligning with classical physisorption behavior of CO_2_ in slit-pore carbons.^23,24^

Finally, the slight reduction of *Q*_st_ in ultrasonicated *vs.* non-US samples (Fig. 9c and d) suggests that some high-energy adsorption sites, potentially copper or N-rich defects, are partially removed or redistributed during cavitation and subsequent washing, even as total capacity increases due to greater microporosity. We retain this nuance in interpreting the net effect: ultrasonication enhances capacity mainly *via* pore-structure exposure/creation, not *via* stronger site energetics.

### 3.5. Application potential for abandoned-mine storage: scope and limitations

The conceptual link to CO_2_ storage in abandoned mines rests on combining adsorptive pre-capture (in FR-4-derived porous carbons) with subsurface sequestration volumes that can be substantial.^19^ In this work, we present material-level performance increases and a process-compatible post-treatment (ultrasonication) but we do not yet provide cycling stability, regeneration energy analysis, nor mine-scale process modeling. The potential application is therefore framed as forward-looking and we have identified the necessary next steps: (i) adsorption–desorption cycling with PEI-free and optimized-PEI materials to assess capacity retention and amine stability; (ii) mass and energy balances for FR-4 feedstocks, including solvent/water usage and amine impregnation footprints; (iii) system integration (beds, flow rates, breakthrough curves) with validated simulations at mine-relevant scale; and (iv) impurity tolerance studies (*e.g.*, moisture, SOx/NOx) pertinent to real gas streams. These additions will convert the current material advance into a feasibility sustainability case.

## 4. Conclusion

In this study we report an FR-4 type PCB waste valorization route that couples one-step KOH/urea activation with post-treatments tailored to the composite microstructure. We hypothesized that the ultrasonication treatment would increase the surface area of the remaining glass fibers by increasing the surface roughness and decreasing the average particle size and the PEI treatment would functionalize the glass fibers, increasing their affinity for CO_2_. Ultrasonication is a robust physical method that fragments glass-fiber/carbon aggregates, increases the surface area and CO_2_ adsorption capacity. PEI functionalization, while increasing affinity, counterintuitively reduces net capacity due to pore blocking in this FR-4-derived porous network. It is possible that lower PEI ratios could provide enough additional CO_2_ affinity without compromising total surface area too much based on other studies.^36^ However, it is likely that the pore structure of the carbon itself prevents effective incorporation into the carbon without pore blocking. It could be possible to better utilize PEI if the carbon pore structure could be modified. Otherwise, a different functionalization molecule which could preferentially functionalize the glass fibers while preserving the carbon pores structure would be best to enhance the CO_2_ capacity of porous carbons produced form PCB waste.

The non-PEI-functionalized sample which was ultrasonicated saw an increase in BET surface area from 444 to 928 m^2^ g^−1^. This corresponded to a similar increase in CO_2_ adsorption capacity from 1.34 to 1.90 mmol g^−1^. This increase in surface area seems to mainly come from a decrease in particle size in both the carbon and glass fiber particles as evidenced by Ar adsorption data and SEM images. The results do not enable quantification of how much the changes in each type of material present in the samples contributes. However, we can conclude that in general ultrasonication improves the adsorption properties of porous carbons made from PCB waste for use in CO_2_ sequestration. PEI, on the other hand, while reducing overall surface area, did notably increase the affinity for CO_2_ with increasing amounts of PEI. Therefore, it is suggested that PEI does assist in enhancing the ability of the porous carbon to adsorb CO_2_, but at the same time reduces the overall surface area by blocking pores within the carbon causing a net reduction in CO_2_ adsorption capacity. It is possible that even lower amounts of PEI functionalization could lead to a net improvement by limiting pore blocking while simultaneously increasing the affinity of the glass fibers for CO_2_.

Finally, we acknowledge that this work remains primarily at the material scale. Quantitative nitrogen incorporation (*e.g.* XPS or CHN analysis), adsorption–desorption cycling, and process modeling for abandoned mine storage were not included and will be essential for future studies. These steps, along with optimization of PEI loading or alternative amine chemistries, will be critical to translate the observed improvements into practical, sustainable CO_2_ capture systems.

## Conflicts of interest

There are no conflicts to declare.

## Supplementary Material

RA-016-D6RA00139D-s001

## Data Availability

The data for this work can be found in the supplementary file and in the data repository: https://doi.org/10.48804/OOGXJM. Supplementary information (SI) is available. See DOI: https://doi.org/10.1039/d6ra00139d.

## References

[cit1] Li J. and Zeng X., Recycling printed circuit boards, in Waste Electr. Electron. Equip. WEEE Handb., Elsevier, 2012, pp. 287–311, doi: 10.1533/9780857096333.3.287

[cit2] Huang K., Guo J., Xu Z. (2009). Recycling of waste printed circuit boards: A review of current technologies and treatment status in China. J. Hazard. Mater..

[cit3] Heidarinejad Z., Dehghani M. H., Heidari M., Javedan G., Ali I., Sillanpää M. (2020). Methods for preparation and activation of activated carbon: a review. Environ. Chem. Lett..

[cit4] Ning C., Lin C. S. K., Hui D. C. W., McKay G. (2017). Waste Printed Circuit Board (PCB) Recycling Techniques. Top. Curr. Chem..

[cit5] Birloaga I., Coman V., Kopacek B., Vegliò F. (2014). An advanced study on the hydrometallurgical processing of waste computer printed circuit boards to extract their valuable content of metals. Waste Manag..

[cit6] Rajagopal R. R., Rajarao R., Cholake S. T., Sahajwalla V. (2017). Sustainable composite panels from non-metallic waste printed circuit boards and automotive plastics. J. Clean. Prod..

[cit7] Ning Y., Li Y., Yuan W., Song X., Guan C., Huang Q. (2025). Preparation and characterization of PA66 composites by waste printed circuit board non-metallic fractions. Polym. Compos..

[cit8] Grigorescu R. M., Ghioca P., Iancu L., David M. E., Ion R.-M., Nicolae C.-A., Gabor R. A., Radu E. R., Ganciarov M., Spurcaciu B., Alexandrescu E., Ciuprina F. (2022). Influence of non-metallic fraction of printed circuit boards waste on recycled polyvinyl chloride from waste wires. J. Appl. Polym. Sci..

[cit9] Selvaraj T., T S., Kaliyavaradhan S. K., Kakria K., Malladi R. C. (2022). Use of E-Waste in Metakaolin Blended Cement Concrete for Sustainable Construction. Sustainability.

[cit10] Zhang S., Wang L., Li B., Gu Y., Wu Y. (2024). Comprehensive evaluation method based on objective and subjective factors: A case of printed circuit board resin waste treatment technologies in China. Resour. Conserv. Recycl..

[cit11] Chien Y.-C., Paul Wang H., Lin K.-S., Huang Y.-J., Yang Y. W. (2000). Fate of bromine in pyrolysis of printed circuit board wastes. Chemosphere.

[cit12] Ke Y., Yang E., Liu X., Liu C., Dong W. (2013). Preparation of porous carbons from non-metallic fractions of waste printed circuit boards by chemical and physical activation. New Carbon Mater..

[cit13] Wu D., Yi M., Zhang D., Zhou J., Yao Z., Wang C., Xu S., Zhou Y., Liu X. (2025). Modification of waste printed circuit board substrates via Fe N doping and their adsorption performance towards tetracyclines antibiotics. J. Water Process Eng..

[cit14] Du X., Wang L., Zhao W., Wang Y., Qi T., Li C. M. (2016). Preparation of hierarchical porous carbon from waste printed circuit boards for high performance electric double-layer capacitors. J. Power Sources.

[cit15] Kan Y., Yue Q., Gao B., Li Q. (2016). Comparison of activated carbons from epoxy resin of waste printed circuit boards with KOH activation by conventional and microwave heating methods. J. Taiwan Inst.
Chem. Eng..

[cit16] Cao X., Shang M., Shang Y., Wei B., Zhang Y., Zhang Y., Kan Y. (2023). Novel hierarchical porous carbons from waste printed circuit boards for oxygen reduction reaction. J. Taiwan Inst. Chem. Eng..

[cit17] Rubel J., Guo X., Madhav D., Verslype S., Zhang J., Ji Y., Eliášová P., Vandeginste V. (2025). Modified porous carbon from waste printed circuit boards for enhanced adsorption of carbon dioxide in abandoned mines. J. Environ. Chem. Eng..

[cit18] Han J., Zhang L., Zhao B., Qin L., Wang Y., Xing F. (2019). The N-doped activated carbon derived from sugarcane bagasse for CO2 adsorption. Ind. Crops Prod..

[cit19] Pan W., Li C., Liang W. (2023). Underground Space Conception Based on Abandoned Mine. Ind. Eng. Innov. Manag..

[cit20] Lunin B. S., Nikolaev A. L. (2023). Effect of Ultrasonic Treatment on the Surface Topography of Quartz Glass. Inorg. Mater..

[cit21] Xu X., Song C., Andresen J. M., Miller B. G., Scaroni A. W. (2002). Novel Polyethylenimine-Modified Mesoporous Molecular Sieve of MCM-41 Type as High-Capacity Adsorbent for CO2 Capture. Energy Fuels.

[cit22] Han Y., Hwang G., Kim H., Haznedaroglu B. Z., Lee B. (2015). Amine-impregnated millimeter-sized spherical silica foams with hierarchical mesoporous–macroporous structure for CO2 capture. Chem. Eng. J..

[cit23] Rouquerol J. , Rouquerol F., Llewellyn P., Maurin G. and Sing K., Adsorption by Powders and Porous Solids: Principles, Methodology and Applications, Academic Press, 2013

[cit24] Thommes M., Kaneko K., Neimark A. V., Olivier J. P., Rodriguez-Reinoso F., Rouquerol J., Sing K. S. W. (2015). Physisorption of gases, with special reference to the evaluation of surface area and pore size distribution (IUPAC Technical Report). Pure Appl. Chem..

[cit25] Cebeci H. H., Yılmaz İ., Yargı Ö., Açıkalın K., Gelir A., Kantürk Figen A. (2023). Upcycling of waste printed circuit boards into metallic pyrolytic carbon for supercapacitor electrode. J. Mater. Sci. Mater. Electron..

[cit26] Kumari S., Avais M., Katiyar J. D., Suman Y. K., Chattopadhyay S. (2022). Polyethylenimine polyampholytes: Synthesis, characterization and dye adsorption study. J. Polym. Res..

[cit27] Wang Y.-Q., Su J., Wu F., Lu P., Yuan L.-F., Yuan W.-E., Sheng J., Jin T. (2012). Biscarbamate cross-linked polyethylenimine derivative with low molecular weight, low cytotoxicity, and high efficiency for gene delivery. Int. J. Nanomedicine.

[cit28] Quan C., Li A., Gao N. (2009). Thermogravimetric analysis and kinetic study on large particles of printed circuit board wastes. Waste Manag..

[cit29] Doebelin N., Kleeberg R. (2015). Profex: a graphical user interface for the Rietveld refinement program BGMN. J. Appl. Crystallogr..

[cit30] Sreńscek-Nazzal J., Kamińska A., Serafin J., Michalkiewicz B. (2024). Chemical Activation of Banana Peel Waste-Derived Biochar Using KOH and Urea for CO2 Capture. Materials.

[cit31] Hsueh Y.-J., Yu C.-C., Lee K.-R., Tseng C.-J., Su B.-J., Wu S.-K., Weng L.-C. (2013). Ordered porous carbon as the catalyst support for proton-exchange membrane fuel cells. Int. J. Hydrog. Energy.

[cit32] Rouquérol F. , Rouquerol J. and Sing K. S. W., Adsorption by Powders and Porous Solids: Principles, Methodology, and Applications, Academic Press, San Diego, 1999

[cit33] Serafin J., Dziejarski B., Sreńscek-Nazzal J. (2023). An innovative and environmentally friendly bioorganic synthesis of activated carbon based on olive stones and its potential application for CO2 capture. Sustain. Mater. Technol..

[cit34] Gautam J. S., Vikram S., Dziejarski B., Sahoo S. (2023). An environmentally friendly synthesis method of activated carbons based on subabul (*Leucaena leucocephala*) sawdust waste for CO2 adsorption. J. Clean. Prod..

[cit35] Wei M., Zhao Q. (2021). CO2 Adsorption and Desorption by Waste Ion-Exchange Resin–Based Activated Carbon on Fixed Bed. Front. Energy Res..

[cit36] Liu H., Lu X., Liu L., Wang J., Wang P., Gao P., Ren T., Tian G., Wang D. (2022). Application of amine-loaded activated carbon fiber in CO2 capture and separation. Korean J. Chem. Eng..

