# Supplementary material for: Transformation of glass fiber reinforced epoxy from waste printed circuit boards to adsorbents for effective CO2 storage in abandoned mines
Source: RSC Adv. 2026 Jan 21;16(5):4655–66. doi: 10.1039/d6ra00139d (PMC12821125; doi:10.1039/d6ra00139d)
Supplement: RA-016-D6RA00139D-s001 [file RA-016-D6RA00139D-s001.pdf]

Supplementary Information (SI) for RSC Advances.
This journal is © The Royal Society of Chemistry 2026

Supplementary Information: Transformation of glass fiber reinforced epoxy from waste printed circuit boards to adsorbents for effective CO_2 storage in abandoned mines

Jacob Rubel[1], Pavla Eliášová[2], Martin Kubů[2], Yukun Ji[3,4,*], Veerle Vandeginste[1,*]

[1] KU Leuven, Campus Brugge, Department of Materials Engineering, 8200 Bruges, Belgium

[2] Charles University, Faculty of Science, Department of Physical and Macromolecular Chemistry, Hlavova 8, Praha 2, 12840, Czech Republic

[3] China University of Mining and Technology, State Key Laboratory for Geomechanics and Deep Underground Engineering, Xuzhou 221116, China

[4] Yunlong Lake Laboratory of Deep Underground Science and Engineering, Xuzhou 221116, China

* Corresponding authors: email: veerle.vandeginste@kuleuven.be (Veerle Vandeginste); jykcumt@163.com (Yukun Ji)

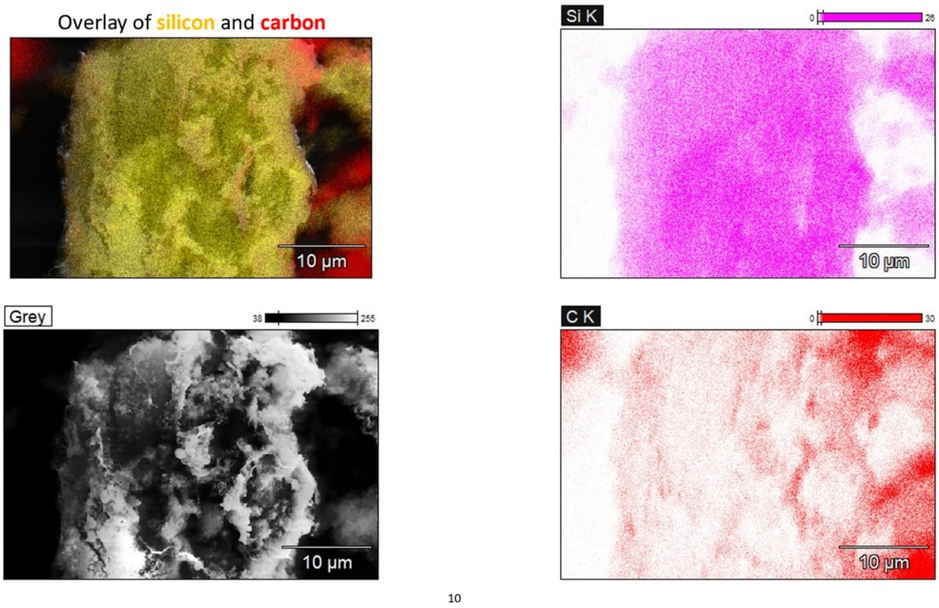

Figure S 1 EDS Mapping of PCB650 for carbon and silicon

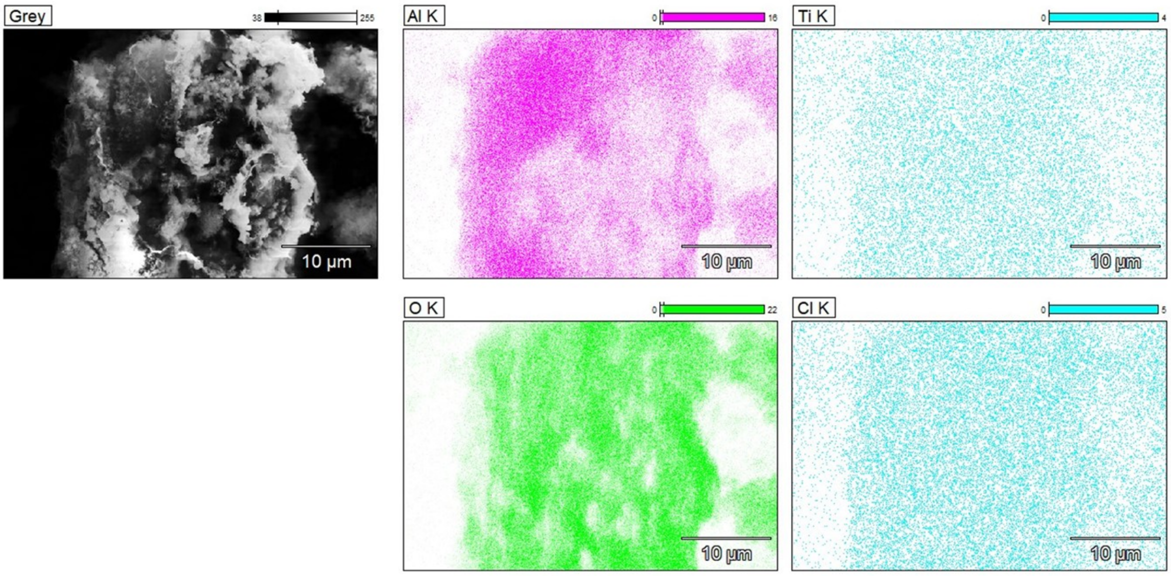

11

Figure S 2EDS Mapping of PCB650 for aluminum, titanium, oxygen, and chlorine

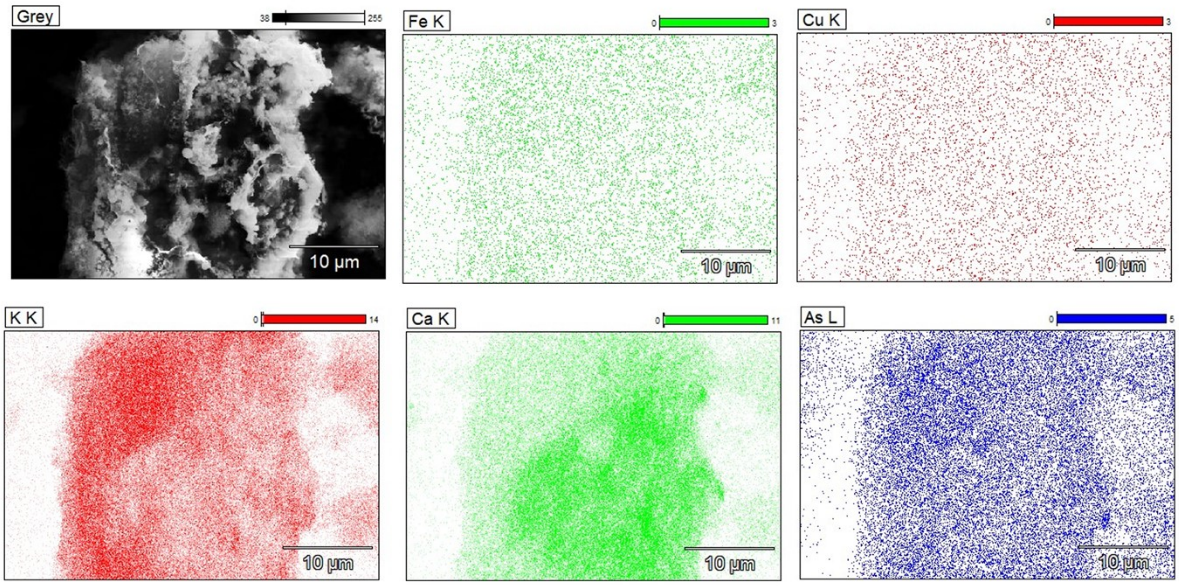

12

Figure S 3 EDS Mapping of PCB650 for iron, copper, potassium, calcium, and arsenic

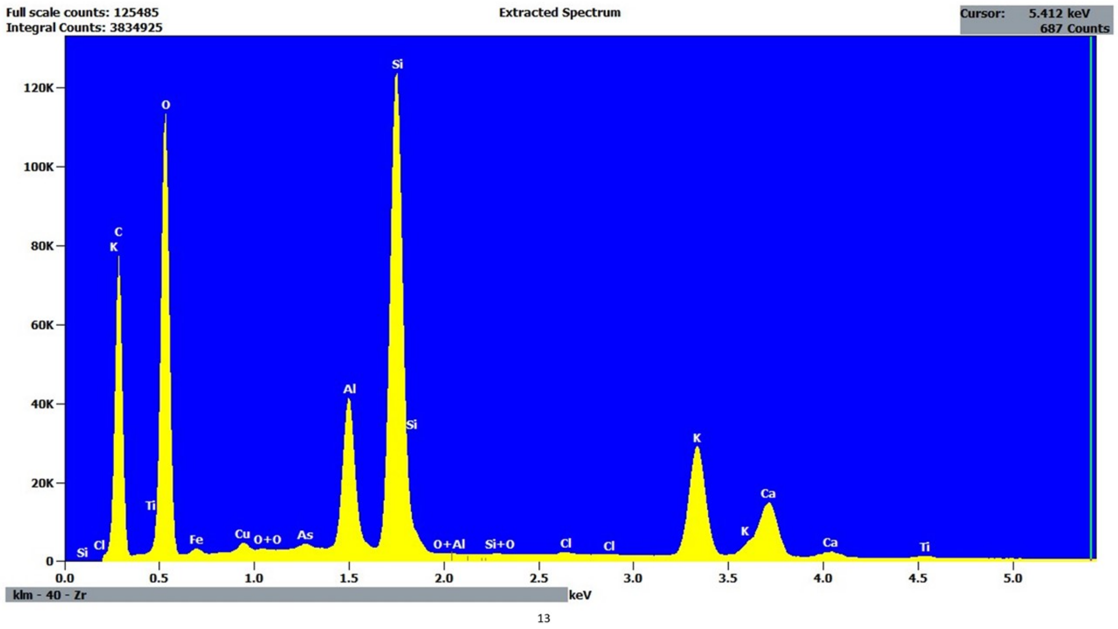

Figure S 4 Elemental spectrum of PCB650

Element	Extracted Spectrum Intensity	Extracted Spectrum Net Counts	Extracted Spectrum Weight %	Extracted Spectrum Atom %	Extracted Spectrum Norm. Wt. %
**C K**	0.00	395626	22.76	33.62	22.76
**O K**	0.00	578174	42.45	47.07	42.45
**Al K**	0.00	295744	4.50	2.96	4.50
**Si K**	0.00	1038953	16.30	10.29	16.30
**Si L**	0.00	0	0.00	0.00	0.00
**Cl K**	0.00	8741	0.17	0.09	0.17
**Cl L**	0.00	3323	0.00	0.00	0.00
**K K**	0.00	325394	7.71	3.50	7.71
**K L**	0.00	0	0.00	0.00	0.00
**Ca K**	0.00	165580	4.53	2.01	4.53
**Ca L**	0.00	0	0.00	0.00	0.00
**Ti K**	0.00	6361	0.26	0.10	0.26
**Ti L**	0.00	15367	0.00	0.00	0.00
**Fe K**	0.00	4312	0.36	0.11	0.36
**Fe L**	0.00	23974	0.00	0.00	0.00
**Cu K**	0.00	4150	0.74	0.21	0.74
**Cu L**	0.00	11065	0.00	0.00	0.00
**As K**	0.00	21	0.00	0.00	0.00
**As L**	0.00	7841	0.21	0.05	0.21
			100.00	100.00	100.00

Table S 1 elemental data for PCB650

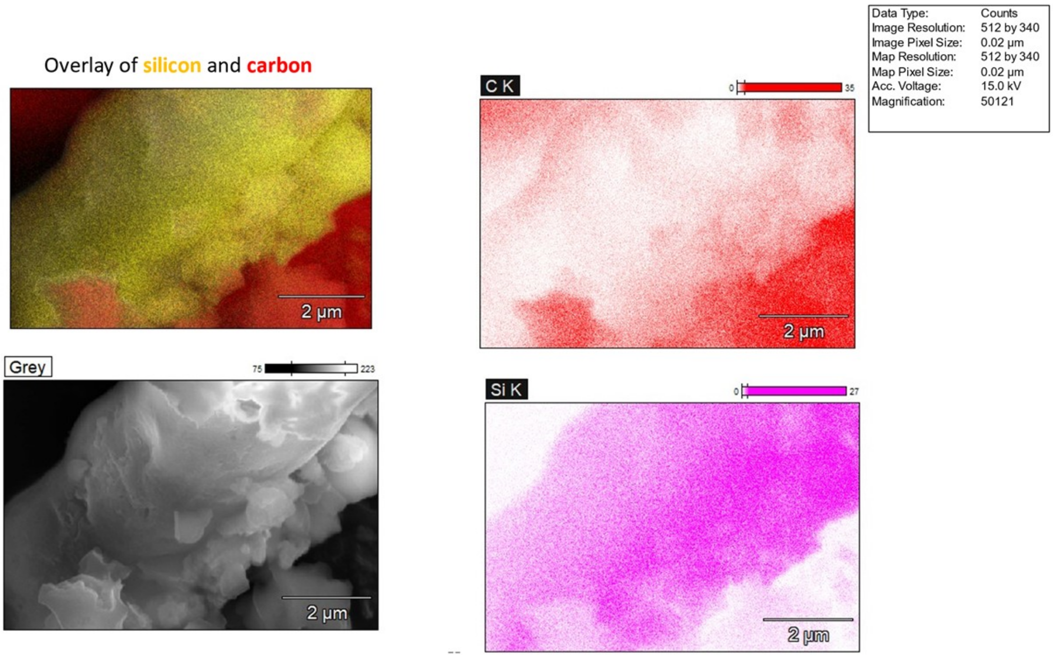

Figure S 5 EDS Mapping of US-PCB650 for carbon and silicon

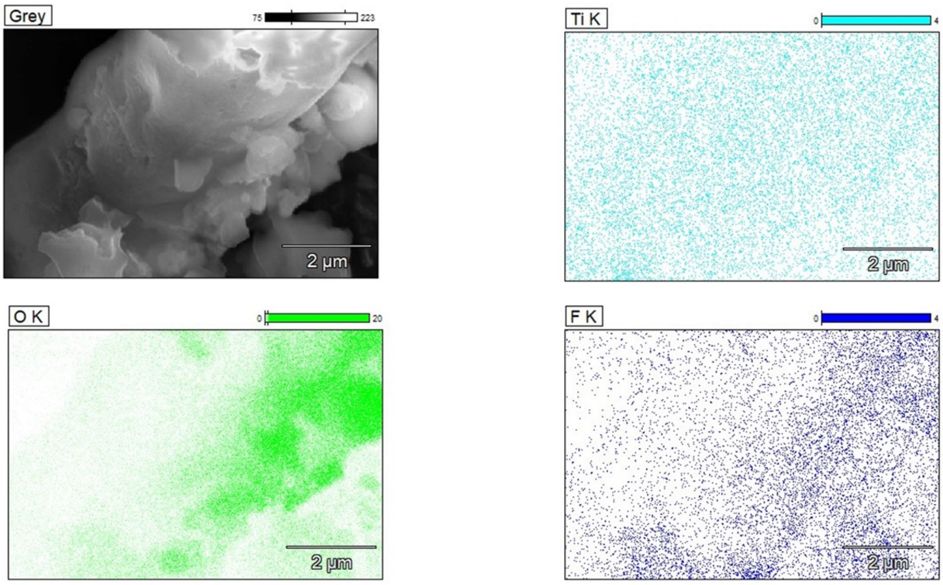

22

Figure S 6 EDS Mapping of US-PCB650 for titanium, oxygen, and fluorine

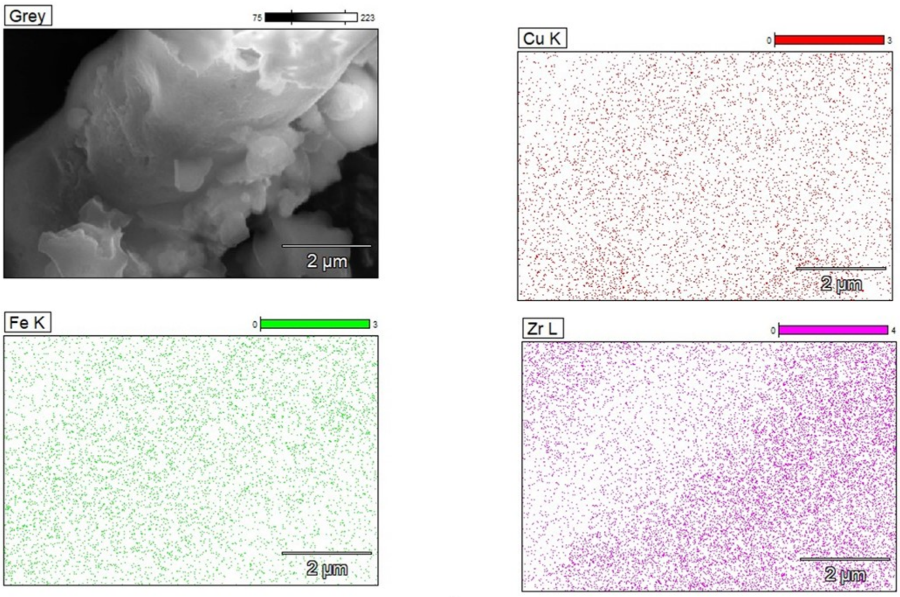

23

Figure S 7 EDS Mapping of US-PCB650 for copper, iron, and zirconium

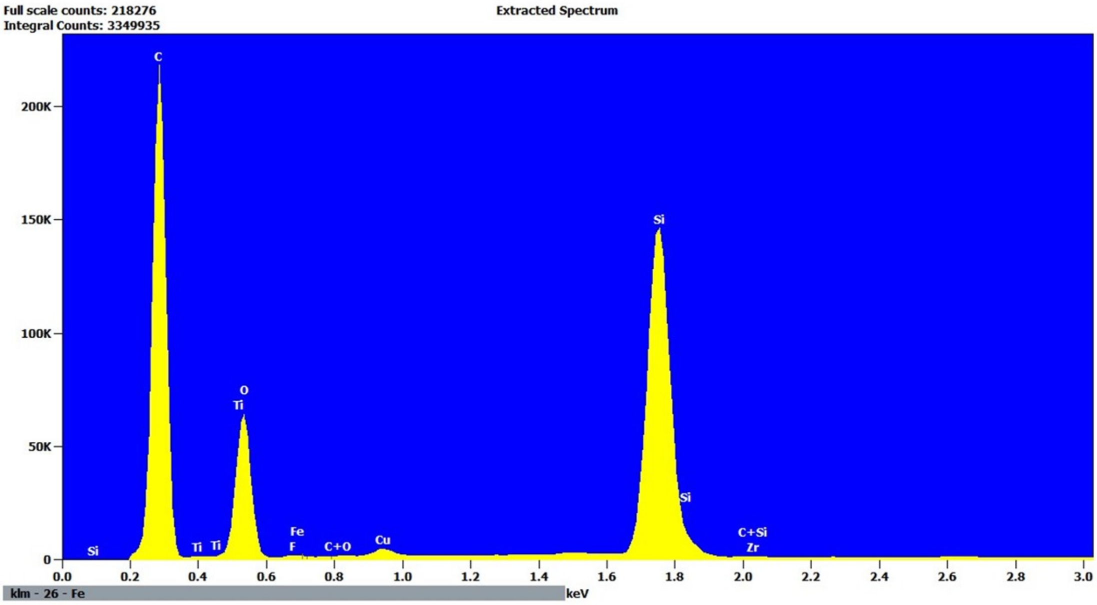

25

Figure S 8 Elemental spectrum of US-PCB650

Element	Extracted Spectrum	Extracted Spectrum	Extracted Spectrum	Extracted Spectrum	Extracted Spectrum
	Intensity	Net Counts	Weight %	Atom %	Norm. Wt. %
**C K**	0.00	1090312	52.91	64.70	52.91
**O K**	0.00	326674	27.97	25.68	27.97
**F K**	0.00	0	0.00	0.00	0.00
**Si K**	0.00	1237997	17.79	9.31	17.79
**Si L**	0.00	0	0.00	0.00	0.00
**Ti K**	0.00	7166	0.29	0.09	0.29
**Ti L**	0.00	97573	0.00	0.00	0.00
**Fe K**	0.00	855	0.07	0.02	0.07
**Fe L**	0.00	12468	0.00	0.00	0.00
**Cu K**	0.00	4399	0.79	0.18	0.79
**Cu L**	0.00	19879	0.00	0.00	0.00
**Zr L**	0.00	5935	0.17	0.03	0.17
**Zr M**	0.00	6556	0.00	0.00	0.00
			100.00	100.00	100.00

Figure S 9 Elemental data for US-PCB650
